# Use of a recombinant *Salmonella enterica *serovar Typhimurium strain expressing C-Raf for protection against C-Raf induced lung adenoma in mice

**DOI:** 10.1186/1471-2407-5-15

**Published:** 2005-02-09

**Authors:** Ivaylo Gentschev, Joachim Fensterle, Andreas Schmidt, Tamara Potapenko, Jakob Troppmair, Werner Goebel, Ulf R Rapp

**Affiliations:** 1 Institut für Medizinische Strahlenkunde und Zellforschung (MSZ), University of Wuerzburg, D-97078 Wuerzburg, Germany; 2 Daniel-Swarovski-Research Laboratory, Department of General and Transplant Surgery, Innsbruck Medical University, Innsbruck, Austria; 3 Department of Microbiology, University of Wuerzburg, D-97074 Wuerzburg, Germany

## Abstract

**Background:**

Serine-threonine kinases of the Raf family (A-Raf, B-Raf, C-Raf) are central players in cellular signal transduction, and thus often causally involved in the development of cancer when mutated or over-expressed. Therefore these proteins are potential targets for immunotherapy and a possible basis for vaccine development against tumors. In this study we analyzed the functionality of a new live C-Raf vaccine based on an attenuated *Salmonella enterica *serovar Typhimurium *aroA *strain in two Raf dependent lung tumor mouse models.

**Methods:**

The antigen C-Raf has been fused to the C-terminal secretion signal of *Escherichia coli *α-hemolysin and expressed in secreted form by an attenuated *aroA Salmonella enterica *serovar Typhimurium strain via the α-hemolysin secretion pathway. The effect of the immunization with this recombinant C-Raf strain on wild-type C57BL/6 or lung tumor bearing transgenic BxB mice was analyzed using western blot and FACS analysis as well as specific tumor growth assays.

**Results:**

C-Raf antigen was successfully expressed in secreted form by an attenuated *Salmonella enterica *serovar Typhimurium *aroA *strain using the *E. coli *hemolysin secretion system. Immunization of wild-type C57BL/6 or tumor bearing mice provoked specific C-Raf antibody and T-cell responses. Most importantly, the vaccine strain significantly reduced tumor growth in two transgenic mouse models of Raf oncogene-induced lung adenomas.

**Conclusions:**

The combination of the C-Raf antigen, hemolysin secretion system and *Salmonella enterica *serovar Typhimurium could form the basis for a new generation of live bacterial vaccines for the treatment of Raf dependent human malignancies.

## Background

The Raf proteins (A-Raf, B-Raf, C-Raf) are located upstream of MEK and downstream of Ras and represent an essential part of the mitogenic cascade [1-3]. Interestingly, Raf kinases are not only central players in cellular signal transduction, but are often causally involved in the development of cancer. Recently, B-Raf was found to be mutated in a broad range of malignancies including melanoma (more than 65%), and colon cancer [4]. In addition, overexpression of C-Raf was found in many tumors [5,6]. Therefore, these proteins are potential targets for immunotherapy as well as immunoprevention of tumors.

Here we describe the development of a C-Raf vaccine on the basis of an attenuated *Salmonella enterica *serovar Typhimurium *aroA *strain. Such recombinant live vaccines have been shown to efficiently elicit both, humoral and cellular immune responses against a variety of heterologous antigens [7,8]. In order to achieve stable expression of C-Raf we used the *E. coli *α-hemolysin (HlyA) secretion system which is fully active in *Salmonella *[9]. This transport machinery is the prototype of type I secretion systems and consists of three different components, namely HlyB, HlyD and TolC. The HlyA carries at its C-terminus a secretion signal of about 50–60 amino acids in length (HlyA_s_), which is recognized by the HlyB/HlyD/TolC-translocator, leading to direct secretion of the entire protein into the extracellular medium without the formation of periplasmic intermediates. In addition, fused to the C-terminus of heterologous proteins, the HlyA_s _leads to efficient secretion of such proteins by the recombinant bacteria. In our case, the whole C-Raf antigen fused to the C-terminal secretion signal of hemolysin was efficiently expressed and secreted by an attenuated *Salmonella enterica *serovar Typhimurium *aroA *strain. The effects of this recombinant vaccine were assessed in wild-type C57BL/6 or tumor bearing transgenic BxB mice.

## Methods

### Bacterial strains, plasmids, cell lines and mice

The bacterial strains, plasmids, cell lines and mice used in this study are listed in Table 1.

### Plasmid transformation in *Salmonella enterica *serovar Typhimurium SL7207

The plasmids pMOhly1 and pMOhly-Raf were first transformed in competent *Salmonella enterica *serovar Typhimurium LB5000 (Tab. 1), a restriction-negative and modification-proficient strain by a standard transformation protocol for *E. coli *[10,11]. Subsequently, the plasmids purified from *Salmonella enterica *serovar Typhimurium LB5000 were introduced into *Salmonella enterica *serovar Typhimurium SL7207 by electroporation using a Bio-Rad Gene Pulser (Hercules, CA, USA) at 2.5 kV, 25 microfarads (μF), and 200 Ohm in a 0.1 cm electroporation cuvette.

### Construction of the plasmid pMOhly-Raf

Sense primer 5' Raf: 5'-ATGGAGCACATACATGCATCTTGGAAG-3' and antisense primer 3'Raf: 5'-CAACTAGAATGCATGCAGCCTCGGGGA-3' were used to amplify by PCR a 1950 bp DNA fragment representing the entire *craf *gene from plasmid pUC13-c-raf-1 [12]. PCRs were performed in a Thermal Cycler 60 (Biometra, Göttingen, Germany) for 30 cycles of 94°C for 1 min, 55°C for 1 min, and 72°C for 90 s. After purification with the GeneClean Kit (Bio101, La Jolla, Ca) and digestion with the *NsiI *restriction enzyme, the DNA fragment carrying the *craf *gene was inserted into the single *NsiI *site of the export vector pMOhly1 [13]. The resulting plasmid pMOhly-Raf was isolated from *E. coli *DH5α (Invitrogen), analyzed and transformed in *S. typhimurium *SL7207 (Tab. 1) by electroporation.

### Western blot analysis

*Salmonella enterica *serovar Typhimurium strains harbouring the recombinant plasmids pMOhly1 or pMOhly-Raf were cultivated at 37°C in BHI medium with 100 μg/ml ampicillin. Cells at the exponential growth phase (OD_600 _of 1) were centrifuged at 5000 × g at 4°C for 5 min. The supernatant proteins were precipitated with 10% (V/V) trichloroacetic acid (TCA) for 1 h on ice, collected by centrifugation and resuspended in SDS sample buffer. The supernatant proteins were separated on 10% gels by SDS-PAGE [14]. For immunodetection of Raf-HlyA proteins the rabbit polyclonal anti-Raf antibody SP-63 (diluted 1:1,000, Rapp Laboratory) and donkey anti-rabbit immunoglobulins linked to horseradish peroxidase (Amersham Pharmacia Biotech) diluted 1:1,000 as secondary antibodies were used. Blots were developed by enhanced chemiluminescence (ECL reagents; Amersham Biosciences, UK) and exposed on X-ray film (Kodak, XO-MAT-AR) for 1 min.

### Immunization of mice with *Salmonella enterica *serovar Typhimurium SL7207 strains

#### Oral/intravenous prime boost protocol (p.o./i.v.)

In order to achieve a broad immune response encompassing both, the mucosal and systemic immunity against C-Raf, we combined oral immunization (p.o.) with an intravenous (i.v.) boost. In these experiments, seven weeks old C57BL/6 or BxB23 mice were immunized, first p.o. three times with 5 × 10^9 ^bacteria/100 μl phosphate-bufferd saline (PBS) at 5-day intervals. At day 45 after the start of the vaccination, these mice were boosted intravenously (i.v.) with a single-dose of 5 × 10^5 ^bacteria/100 μl PBS. The BxB23 mice received a second i.v. boost of 5 × 10^5 ^bacteria at day 90 after the start of the vaccination. Induction of Raf-specific immune responses were analyzed at day 50 for C57BL/6 or at day 95 for BxB23 respectively.

#### Intranasal Immunization (i.n.)

Seven weeks old BxB23 mice were immunized i.n. four times with 1 × 10^7^bacteria/30 μl phosphate-buffered saline (PBS) at 14-day intervals. The vaccine was applied using a micropipette onto the nares of mice under anesthesia.

Induction of Raf-specific immune responses were analyzed at day 70.

#### I. n. Immunization of BXB11 mice with Salmonella enterica serovar Typhimurium SL7207/pMOhly-Raf

Four months old BXB11 mice were immunized i.n. three times with 1 × 10^8^salmonellae in 10 μl PBS at 14-day intervals. The vaccine was applied using a micropipette into the nares of mice without anesthesia.

### Construction of a C-Raf overexpressing EL-4 cell line

In order to create tools for analysis of C-Raf specific T cells we first constructed a C-Raf overexpressing EL-4 cell line (ELRaf). The EL4 tumor cell line is a murine thymoma cell line of the H-2^b ^haplotype (Tab. 1). 10 μg purified DNA of plasmid pDNA3craf was introduced into EL4 cells by electroporation with the Bio-Rad Gene Pulser (Hercules, CA) at 0.25 kV, 960 μFD, in a 0.4-cm electroporation cuvette. The transformed cells were grown RPMI 1640 medium (Invitrogen) supplemented with 5% (vol/vol) FCS (PAN Systems) and 600 μg/ml G418 (Sigma). C-Raf is highly expressed in ELRaf cells (data not shown).

### Flow cytometric detection of specific CD8^+ ^T-cells

#### Isolation of spleen cells

Animals were sacrificed and a single cell suspension of splenocytes was prepared by passage of the spleen through a sieve into RP 10 medium [RPMI medium (Life Technologies) supplemented with glutamine (1%), 50 μM β-mercaptoethanol (ROTH, Wuerzburg), penicillin (10 U/ml, GIBGO), streptomycin (100 U/ml, GIBGO) and 10% fetal calf serum (PAN™, BIOTECH GmbH).

The cell suspension was centrifuged and resuspended in 3 ml lysis buffer (5 mM Tris-HCl, 140 mM NH_4_Cl, pH 7.3) for the lysis of erythrocytes. After 2 minutes, 10 ml RP 10 medium was added to stop lysis. After centrifugation, cells were resuspended in 2 ml RP 10 medium and counted.

#### Restimulation

In 6 ml FACS tubes and a total volume of 100 μl RP 10 medium in the presence of 30 U/ml recombinant IL-2 and 10 μg/ml Brefeldin A at 37°C, 5% CO_2_, 1 × 10^6 ^spleen cells were incubated for 5 hours with 5 × 10^5 ^C-Raf overexpressing EL-4 cells (EL-Raf) or 5 × 10^5 ^EL-4 cells or with the addition of 10 ng/ml of phorbol myristyl acetate (PMA; Sigma) and 500 ng/ml of ionomycin (Sigma) or with medium alone.

#### Staining

After restimulation, cells were washed with PBS-0.1% bovine serum albumin (BSA; P-B buffer) and incubated with 1 μl of anti-CD8-CyChrome (Pharmingen Nr. 01082A) in a volume of 100 μl PBS-0.1% BSA for 20 min on ice. Subsequently, cells were washed and fixed for 20 min at room temperature with PBS-2% paraformaldehyde (Sigma). After washing with PBS-0.1% (BSA), cells were permeabilized with PBS-0.1% BSA-0.5% saponin (Sigma, P-B-S) buffer. After incubation for 5 min at room temperature, cells were washed with P-B-S buffer and incubated in a volume of 100 μl at room temperature with polyclonal rat immunoglobulin G (IgG) antibodies (JacksonImmunoResearch) to block nonspecific binding and 1 μl anti-IFN-γ-FITC (AN18.17.24; XMG1.2 Rat IgG1; Pharmingen) for 20 min. After incubation, cells were washed twice with P-B-S buffer and another time with P-B buffer. Cells were resuspended in 400 μl PBS-PFA 0,5% and kept at 4°C until analysis.

#### Analysis

Cells were analyzed by flow cytometry in a FACS Calibur flow cytometer (Becton Dickinson) using CellQuest 3.0 software (Becton Dickinson). Lymphocytes were chosen according to their size and granularity in a forward/side – scatter diagram. Numbers are expressed as percent IFN-γ positive CD8^+ ^cells.

### Statistical analysis

The statistical significance of differential findings between experimental groups was determined by Student's *t *test. Findings were regarded as significant, if *P *values were <0.05. Survival curves were compared using a log rank test.

## Results

### Creation of a C-Raf vaccine on the basis of the attenuated *Salmonella enterica *serovar Typhimurium strain SL7207

**Figure 1:**
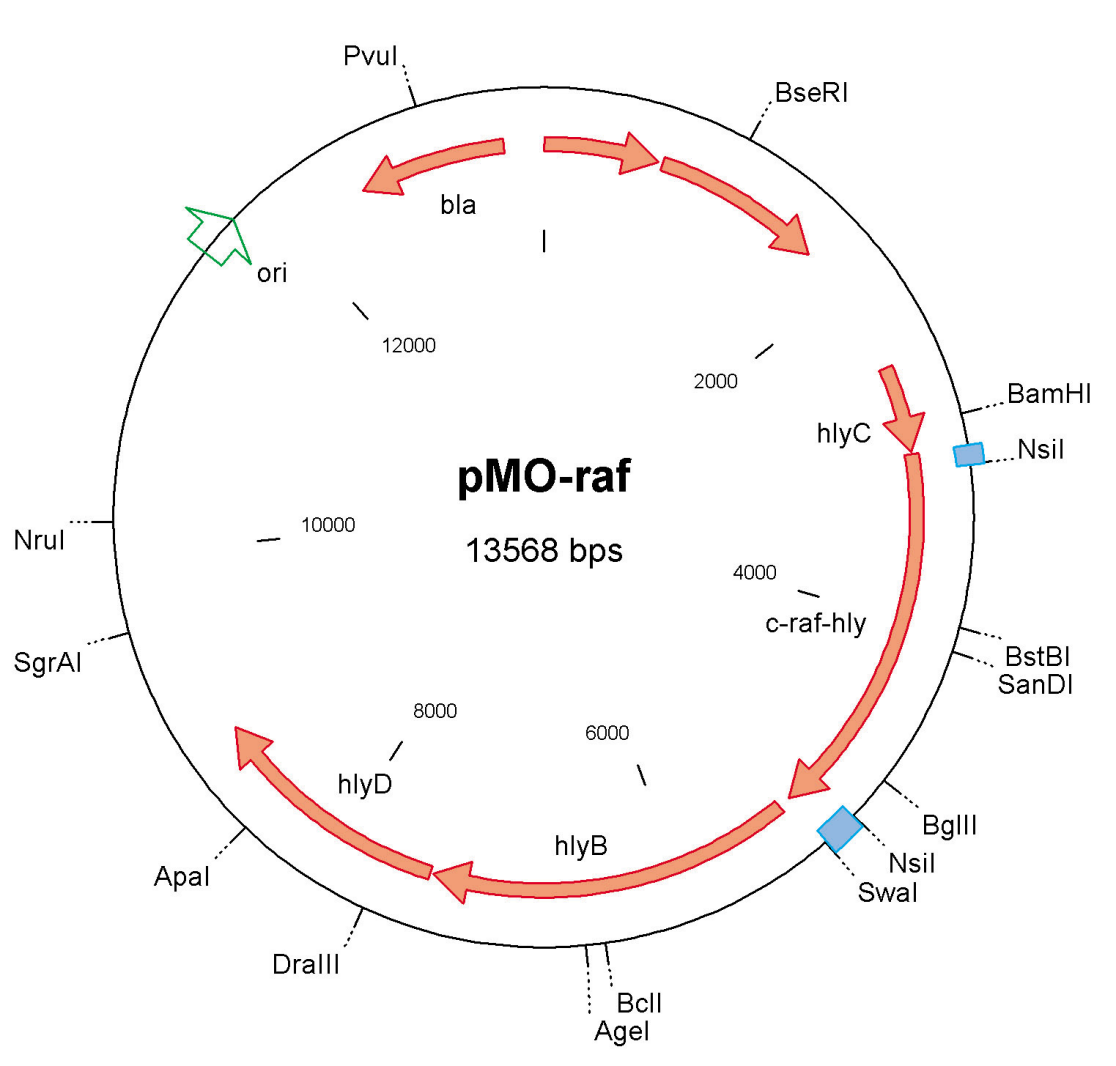
Restriction map of plasmid pMOhly-Raf The plasmid pMOhly-Raf contains the intact structural genes *hlyC*, *hlyB *and *hlyD *of the hemolysin operon and the *c-raf-hlyA *fusion hybrid gene. All genes are transcribed from the original cis-acting expression sites in front of *hlyC *[27]. Abbreviations: ori – origin of replication, bla – ampicillin resistance cassette

**Figure 2:**
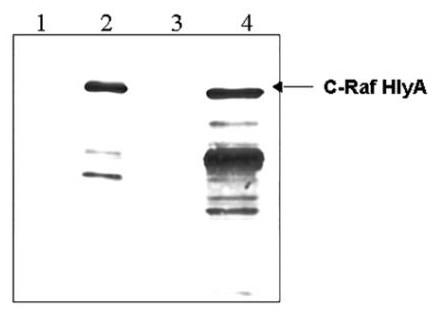
Identification of the Raf-HlyAs fusion protein by immunoblotting. Cultures of *Salmonella enterica *serovar Typhimurium SL7207 carrying the plasmids pMOhly1 (lanes 1 and 3) or pMOhly-Raf (lanes 2 and 4) were grown in BHI medium to a density of 5 × 10^8 ^cells per ml (optical cell density OD600 = 1). Supernatant proteins precipitated from 1.5 ml of bacterial culture were loaded in lanes 1 and 2; cellular proteins from 0.15 ml of culture were loaded in lanes 3 and 4. The immunoblot was developed with polyclonal anti C-Raf antibodies. The samplers were prepared as described in Materials and Methods.

The construction of the attenuated *Salmonella enterica *serovar Typhimurium *aroA *strain SL7207 secreting the C-Raf antigen was achieved by cloning the human *craf *cDNA from pUC13-c-raf-1 [12] into the vector plasmid pMOhly1 [13] as described in materials and methods. The resulting plasmid pMOhly-Raf carried the *craf-hlyAs *fused gene and the functional *hlyB *and *hlyD *genes required for its secretion (Fig. 1). The *S. typhimurium *SL7207/pMOhly-Raf strain efficiently expressed and secreted the hybrid C-Raf protein, as shown by immunoblotting with polyclonal antibodies raised against C-Raf (Fig. 2). The amount of secreted C-Raf was 2–3 μg protein/ml supernatant under the experimental conditions.

### Raf-specific responses of mice after immunization with recombinant SL7207/pMOhly-Raf

**Figure 3:**
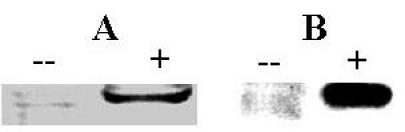
Raf-specific IgG in sera of i.n (A) or p.o/i.v. (B) immunized BxB23 mice (serum dilution 1:200) demonstrated by western blotting. Proteins of 10^6 ^lysed SF9 cells expressing recombinant C-Raf [28] (lanes marked with +) or proteins of 10^6 ^lysed SF9 cells (– lanes) as control were loaded per lane.

The efficacy of the recombinant bacterial strain to induce a Raf-specific immune response was analyzed using wild-type C57BL/6 and transgenic BxB23 mice. Groups of five C57BL/6 and BxB23 mice at the age of 7 weeks were immunized p.o./i.v or i.n. with recombinant *Salmonella enterica *serovar Typhimurium SL7207 secreting C-Raf antigen (SL7207/pMOhly-Raf) and with *Salmonella enterica *serovar Typhimurium SL7207 as control in order to test the induction of Raf-specific immune responses. The data showed that 20% of the sera of BxB23 mice immunized with SL7207/pMOhly-Raf contained Raf-specific antibodies (Fig. 3; supplementary Fig. 1 [see Additional file 1]). In contrast, no Raf-specific IgG response was detectable in the sera of BxB23 mice immunized with SL7207 alone (data not shown). Similar data were obtained after immunization of C57BL/6 mice with SL7207/pMOhly-Raf and SL7207 (data not shown).

**Figure 4:**
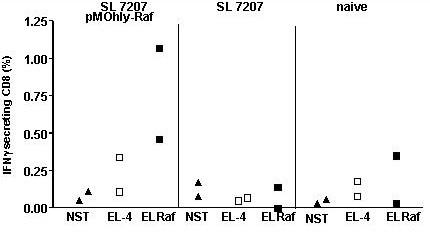
Increased frequency of IFN-γ-secreting CD8^+ ^T cells after p.o./i.v. *Salmonella *infection of C57BL/6 mice (n = 3). For each group, 1 mouse was assessed individually and the remaining splenocytes from 2 mice were pooled in an equivalent proportion. 1 × 10^6 ^spleen cells restimulated with either wild-type EL-4 (EL-4) or C-Raf overexpressing EL-4 cell (EL-Raf) and not stimulated (NST) were used. Production of IFN-γ was determined by flow cytometry after CD8 surface staining and intracellular IFN-γ staining. Each data point represents the proportion of IFN-γ positive cells for one individual mouse or two pooled mice respectively. Intracellular staining with an FITC-conjugated isotype control mAb always resulted in <0.05% positive cells.

To assess the induction of Raf-specific T-cell responses, mice were sacrificed and Raf specific T-cell responses were assessed using intracellular IFN-γ staining followed by FACS analysis. For this purpose, T-cells were restimulated with C-Raf overexpressing EL-4 cells. Using this technique, we could detect Raf specific CD8^+ ^T-cell response in C57BL/6 animals immunized p.o./i.v. with SL7207/pMOhly-Raf only but not in mice immunized i.n. or with the control strain SL7207 (Fig. 4). In immunized BxB23 mice, but not naïve BxB23 mice, a high background and variability of CD8^+ ^IFN-γ positive cells even with non-specific stimulation was observed in two independent experiments. Therefore it was not possible to assess specific T-cell responses in this setting. Interestingly, the level of T-cells which responded to polyclonal stimulation by PMA / ionomycin was also reduced about 4 fold in comparison to C57BL/6 mice (data not shown).

### *Salmonella enterica *serovar Typhimurium SL7207/pMOhly-Raf strain induced partial protection against lung cancer in transgenic mouse models of Raf oncogene-induced lung adenomas

**Figure 5:**
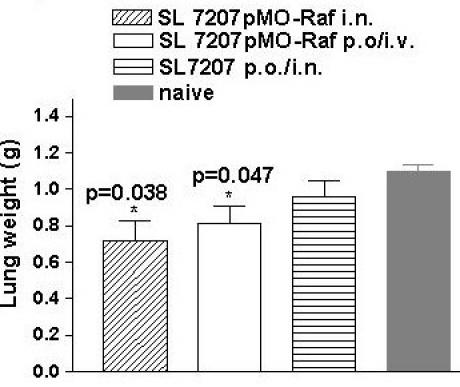
Effect of immunization on reduction of lung weight. Reduction of lung weight is a mark for a delayed tumor growth. Lung weight of 12–13-month-old BxB23 mice immunized with SL7207/pMO-Raf (i.n., n = 10), SL7207/pMO Raf (p.o./i.v., n = 8), SL7207 (i.n or p.o./i.v., n = 6) and naive (n = 6). Bars represent means and standard deviation of the mice per group. Differences in lung weight (tumor weight) between experimental groups treated with SL7207pMO-Raf (i.n and p.o./i.v.) and all control groups were statistically significant * (P < 0.05), as determined by Student's t test. n – number of the mice

To test the protective capacity of the immune responses induced by the recombinant *Salmonella *strains, groups of 6 to 10 heterozygous BxB23 mice at the age of 7 weeks were immunized p.o./i.v. or i.n. with recombinant *Salmonella enterica *serovar Typhimurium strain (SL7207/pMOhly-Raf) or with SL7207 as control. BxB23 mice normally show an induction of lung adenomas with short latency and at 100% incidence [15,16]. The development of lung adenomas in the vaccinated BxB23 mice was assessed for 13 months. Our analysis revealed a significantly delayed tumor growth (reduction of lung weight) in mice immunized i.n. or p.o./i.v. with SL7207/pMOhly-Raf compared to control mice (Fig. 5).

**Figure 6:**
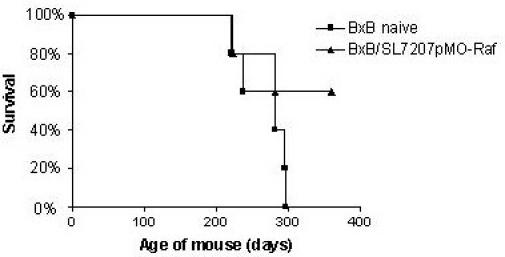
Survival of naive BxB11 (■) or BxB11 mice immunized intranasally with SL7207 pMOhly-Raf (▲) in period of one year.

In order to confirm these data we repeated the protection experiments using BxB11, another *craf *transgenic strain, which develop lung adenomas after a shorter latency period compared to BxB23 mice [15]. In this case, 66% of the mice immunized with SL7207/pMOhly-Raf survived at least three months longer than the control mice (Fig. 6).

These results suggest that the immunization with the vaccine strain SL7207/pMOhly-Raf can achieve a partial protection against tumor growth in the BxB mouse model.

## Discussion

The major problems for vaccine development against cancer are the heterogeneity of the tumor cells and the fact that all tumor antigens are self-antigens. Therefore specific T-cells might be anergic or tolerant [17,18].

However, despite these problems, several cancer vaccines have already reached clinical trials [19]. The success of these vaccines seems to be dependent on the target antigen and on the tumor type.

Here we describe a new strategy for achieving an anti-tumor immune response with a C-Raf vaccine on the basis of an attenuated *Salmonella enterica *serovar Typhimurium *aroA *strain as a "live vaccine". In general, recombinant *aroA *salmonellae are efficient live bacterial vectors that stimulate strong mucosal immunity, humoral and cell-mediated responses with a great potential as live vaccine carriers in both humans and animals [7,20]. Advantages of live attenuated *aroA Salmonella *vaccines include their safety and easy administration [21,22]. In addition *aroA Salmonella enterica *serovar Typhimurium strains were already successfully used as carriers for DNA vaccines against cancer in mice [23].

In this study the C-Raf antigen was delivered in secreted form by attenuated *Salmonella enterica *serovar Typhimurium *aroA *strain using the *E. coli *hemolysin secretion system. This system allows an efficient antigen secretion and presentation, which are necessary for an optimal immune response against the given heterologous antigen [9]. In fact, immunization of mice with the *Salmonella enterica *serovar Typhimurium *aroA *strain SL7207 secreting C-Raf resulted in the induction of a humoral immune response, manifested by the presence of Raf-specific antibodies in some of the vaccinated mice. In addition, C57BL/6 mice immunized with *Salmonella enterica *serovar Typhimurium SL7207/pMOhly-Raf developed a Raf-specific CD8^+ ^T-cell response. The C-Raf vaccine thus is able to break the peripheral tolerance of the immune system towards C-Raf and to induce a specific immune response. Although the transgenic model is closer to the human setting in comparison to challenge models with tumor cell lines, we can not formally exclude that lung specific expression of the transgene in our BxB model is not sufficient for the induction of peripheral tolerance. Furthermore, side effects due to autoimmunity might occur in humans who have a more generalized expression pattern. However, the lung tissue does not belong to immunoprivileged sites and we have never observed any signs of lung pathology in immunized animals which strongly suggests that the data could, in principle, be translated into the human setting.

We demonstrated also a partial tumor protection both in BxB23 and in BxB11 mice after immunization with recombinant *Salmonella enterica *serovar Typhimurium SL7207/pMOhly-Raf. However, we were not able to assess Raf specific CTL responses in these mice due to the high variability and background and therefore we cannot conclude whether protection is really due to cytotoxic T-cells or might be the result of other effects, e.g. an increased intratumoral level of IFN-γ produced by Raf specific CD4^+ ^T-cells. Interestingly, the observed variation occurred only in treated BxB23 mice, not in naïve BxB23 mice. Therefore, the observed effect might be due to a spontaneous induction of an immune response mediated by tumor infiltrating bacteria. It is interesting to note that spontaneous Raf specific immune responses also occur in the human context, which we have recently demonstrated B-Raf V599E specific CTL and B-Raf /B-Raf V599E specific humoral responses in melanoma patients [24,25].

Most importantly, the lack of B-Raf V599E mutations in metastases of melanoma patients with strong B-Raf V599E CD8 response [24] supports the notion that CD8^+ ^T-cells are effective in eliminating antigen positive tumor cells. The combined data thus provide proof of concept for the development of Raf-based anti-cancer vaccines.

This is the first example demonstrating that the *E. coli *hemolysin secretion system is a versatile tool for the delivery of cancer antigens in *Salmonella enterica *serovar Typhimurium. Moreover, we have recently shown that this secretion system is also fully active in *Salmonella enterica *serovar Typhi Ty21a, the only *Salmonella *vaccine strain registered for human use [26]. Therefore, the combination of the hemolysin secretion system and *S. typhi *Ty21a could form the basis for a new generation of live bacterial vaccines against cancer.

## Conclusions

Taken together we have demonstrated that the C-Raf antigen can be successfully expressed and secreted by the attenuated *Salmonella enterica *serovar Typhimurium *aroA *SL7207 strain via the *E. coli *hemolysin secretion system. In addition, the immunization of wild-type C57BL/6 or tumor bearing transgenic mice with this C-Raf secreting *Salmonella *strain provoked specific C-Raf antibody and T cell responses. Most importantly, the vaccine strain induced partial protection against lung cancer in two transgenic mouse models of Raf oncogene-induced lung adenomas.

The approach may provide a new strategy for the rational design of cancer therapies.

## Competing interests

The author(s) declare that they have no competing interests.

## Authors' contributions

IG, JF and JT designed the study. IG drafted the manuscript. JF was also involved in writing the report. AS and JF did the FACS analyses. JT constructed the EL4Raf cell line. TP, AS, JF and IG carried out the immunization of mice and the Western blot analyses. WG and URR were involved in providing the conceptual framework for this study. All authors approved the final version of the manuscript.

## Pre-publication history

The pre-publication history for this paper can be accessed here:



## Supplementary Material

Additional File 1 Supplementary figure 1: C-Raf-specific IgG in sera of p.o/i.v. (A) or i.n (B) immunized BxB23 mice (serum dilution 1:1000) demonstrated by western blotting. Supplementary figure with WESTERN Blot analysis of positive C-Raf sera. Click here for file

## Figures and Tables

**Table 1:** Bacterial strains, plasmids (Ap^R^-ampicillin-resistant), cell lines and mice

Name	Relevant characteristics/sequence	Source or reference
**Bacterial strains:**		
*E. coli *DH5α	F^-^, ø80d*lacZ*ΔM15, Δ(*lacZYA-argF*)U169 *deoR*, *recA*1, *endA*1, *hsdR*17(rk^-^, mk^+^), *phoA*, *supE*44, λ^-^, *thi*-1, *gyrA*96, *relA*1	Takaba
*Salmonella enterica *serovar		
Typhimurium *aroA *SL7207	*his*G46, DEL407 [*aroA*544::Tn10 (Tc^s^)]	Stocker, B. A. D.
*Salmonella enterica *serovar		
Typhimurium LB5000	rk^-^, mk^+^	Stocker, B. A. D.
**Plasmids:**		
pUC13-c-raf-1	*c-raf *cDNA in pUC13	[12]
pcDNA3	pCMV, Ap^R^, Neomycin, SV 40, ColE	Invitrogene
pcDNA-craf	*craf *cDNA in pcDNA3	Troppmair, J
pMOhly1	Ap^R^, *hlyR*, *hlyC*, *hlyAs *(encoding the hemolysin secretion signal), *hlyB*, *hlyD*	[13]
pMOhly-Raf	Ap^R^, *hlyR*, *hlyC*, *raf-hlyAs *(encoding a C-Raf-hemolysin fusion), *hlyB*, *hlyD*	this study
**Cell lines:**		
EL-4	spontaneous murine lymphoma	ATCC(Rockville, MD, USA)
EL-4Raf	pcDNA-craf transfected EL-4 cells	this study
SF9	insect cell line	Gibco
SF9 Raf	*craf *transfected SF9 cells	[28]
**Mice:**		
C57BL/6JolaHsd	wild-type (H-2^b^)	Harlan-Winkelmann, Borche, Germany
BxB23	[(C57BL/6 × DBA-2)F_1_] expressing an oncogenically activated NH_2_-terminal deletion mutant c-Raf-1-BxB under the control of human *SP-C *promoter	[15]
BxB11	[(C57BL/6 × DBA-2)F_1_] expressing an oncogenically activated NH_2_-terminal deletion mutant c-Raf-1-BxB under the control of human *SP-C *promoter	[15]
