# Supplementary material for: Use of a recombinant Salmonella enterica serovar Typhimurium strain expressing C-Raf for protection against C-Raf induced lung adenoma in mice
Source: BMC Cancer. 2005 Feb 9;5:15. doi: 10.1186/1471-2407-5-15 (PMC549196; doi:10.1186/1471-2407-5-15)
Supplement: Additional File 1 — Supplementary figure 1: C-Raf-specific IgG in sera of p.o/i.v. (A) or i.n (B) immunized BxB23 mice (serum dilution 1:1000) demonstrated by western blotting. Supplementary figure with WESTERN Blot analysis of positive C-Raf sera. [file 1471-2407-5-15-S1.pdf]

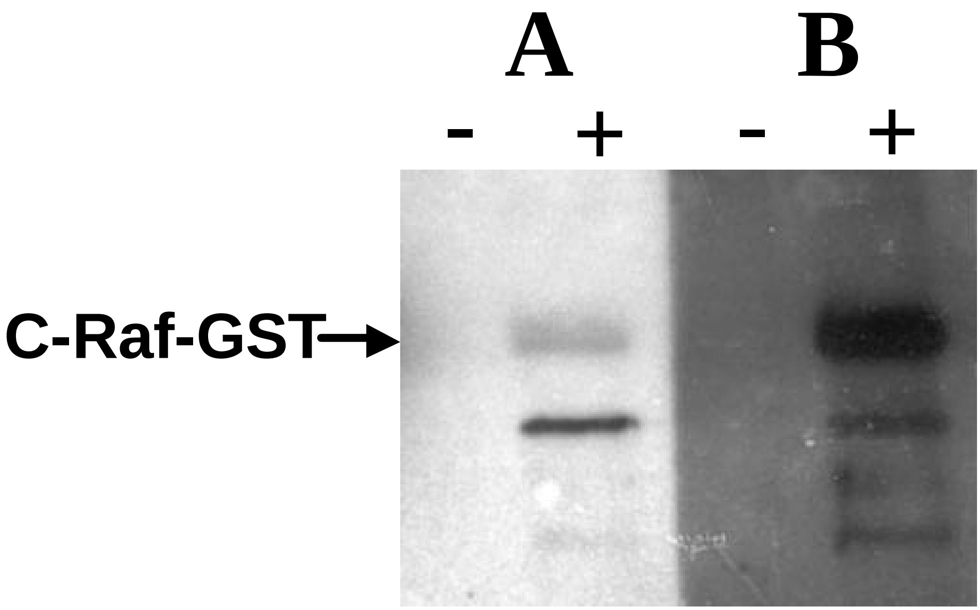

Supplementary figure 1.

C-Raf-specific IgG in sera of p.o/i.v. (A) or i.n (B) immunized BxB23 mice (serum dilution 1:1000) demonstrated by western blotting.

- Lanes: preimmune sera; + lanes: sera of the imminized BxB23 mice. 1 µg purified C-Raf-GST (glutathione *S*-transferase) was loaded per lane. Recombinant wild type C-Raf protein was expressed in Sf9 cells and purified as a GST fusion as described (Hekman M, et al., J Biol Chem 2002; 277:24090–24102). Purity of the C-Raf-GST preparations was controlled by SDS-polyacrylamide gel electrophoresis and staining with Coomassie Blue.
